# Editorial: Diagnosis, prevention and treatment in diabetic nephropathy, volume III

**DOI:** 10.3389/fendo.2025.1586022

**Published:** 2025-03-18

**Authors:** Maria Margherita Rando, Federico Biscetti, Andrea Flex

**Affiliations:** ^1^ Department of Cardiovascular Sciences, Cardiovascular Internal Medicine Unit, Fondazione Policlinico Universitario A. Gemelli IRCCS, Roma, Italy; ^2^ Department of Cardiovascular Sciences, Università Cattolica del Sacro Cuore, Roma, Italy

**Keywords:** diabetes mellitus, kidney, diabetic nephropathy, chronic kidney disease, end-stage kidney disease

Diabetic nephropathy (DN) represents the leading cause of end-stage kidney disease (ESKD), affecting approximately 30-40% of diabetic patients. It is characterized by progressive kidney damage, which manifests with albuminuria, declining glomerular filtration rate (GFR), and structural changes such as glomerulosclerosis and tubulointerstitial fibrosis. Contemporary medical research has made great strides in understanding diabetes mellitus (DM) complications including DN. From new biomarkers to treatments based on nanomedicine and the interaction between gut microbiota and kidney disease, the studies included in this Research Topic offer a comprehensive and multidimensional vision to improve diagnosis, prevention and management of diabetic kidney disease.

Zhang et al. provide an overview of the state of the art on DN pathogenesis and treatment, focusing on connection of DN with cardiovascular conditions like hypertension and coronary artery disease. Thus, a correct and holistic management of this condition is crucial to reduce mortality and improve quality of life.

In this regard, even in this third Research Topic on DN, the emphasis remains strong on the need for innovative biomarkers to facilitate early detection and monitoring disease progression.

Of interest, given the interplay between diabetes and its microvascular and macrovascular complications, several works explored the role of innovative inflammatory biomarker in the setting of DN.

In particular, Jiang et al. found that vascular endothelial growth factor (VEGF) was strongly associated with DN progression and glycemic control in a population of patients with type 2 DM, suggesting a predictive role of VEGF for glycemic control and DN in older adults with type 2 DM.

Nie et al., in a study conducted on the National Health and Nutrition Examination Survey (NHANES) database, showed an association between the systemic immune-inflammation index (SII), an inflammatory biomarker which integrates neutrophils, lymphocytes, and platelets, and higher prevalence of diabetes; specifically, each unit increase in SII/100 increased the likelihood of having diabetes by 4%.

Moreover, in a Chinese population, Yan et al. showed that higher SII is independently associated with an increased risk of DN and its severity, suggesting that SII might be a promising biomarker for DN and its distinct phenotypes in the Chinese population.

Compared to other inflammatory markers, including SII, monocyte-to-lymphocyte ratio (MLR) and systemic inflammatory response index (SIRI), Li et al. showed instead that neutrophil-to-lymphocyte ratio (NLR) seems to be more effective in identifying the risk of DN, albuminuria, and low-eGFR in type 2 DM patients. However, type 2 DM patients with elevated levels of NLR, SII, MLR and SIRI should be closely monitored for their potential risk to renal function.

Another potential prognostic marker for renal endpoint events in individuals with DN, has been explored by Sun et al. which showed that serum secretory leukocyte protein inhibitor (SLPI), a protein with antiprotease, anti-inflammatory and immunomodulatory activity, expressed in distal renal tubular cells was associated with DN progression and clinical parameters of DN.

Among innovative biomarkers, Cao et al. analyzed the role of retinol-binding protein 4 (RBP4) as a marker for the detection of renal impairment, showing its potential as a diagnostic tool with good sensitivity and specificity for patients with type 2 DM and DN.

Even tumoral markers, such as CEA, SCC, and CA211, have been explored in a study by Chen et al. showing a potential in the early detection of microalbuminuria in patients with type 2 DM.

In a study based on 4888 subjects of the National Nutrition and Health Examination Survey (NHANES) database Tang et al. showed instead that higher levels of lactate dehydrogenase (LDH), an easy-to-obtain marker, were an independent risk factor for the risk of DN in patients with type 2 DM.

Interestingly, this Research Topic also presents us with works that explore the correlation between fecal microbiota and DN.

In particular, alterations in the gut bacterial composition and metabolites, including Trimethylamine (TMA) and trimethylamine-N-oxide (TMAO), have been correlated with progression of DN, as showed by the studies of Yu et al. and Huo et al.

Specifically, as described by Yu et al., kidney injury in diabetic patients can enhance dysbiosis of the gut microbiota, which can itself further impair renal function through several metabolites. For this reason, enhancing gut microbiota stability, improving glucose metabolism, and reducing uremic toxin production through dietary adjustments, fecal microbiota transplantation (FMT), and the use of probiotics or prebiotics can help delay the progression of DN.

Moreover, as demonstrated by Lu et al., genetic variants associated with gut microbiota composition can also influence the susceptibility to DN, opening new avenues for personalized approaches.

Another point addressed in this topic includes the relationship between lifestyle and DN.

We already know that obesity and sleep duration are associated with cardiovascular risk factors, including insulin resistance, hyperglycemia, hypertension, dyslipidemia, and inflammatory response. Liu et al., showed that BMI changes and sleep duration were determinants of ND progression. Indeed, excessive sleep and increased BMI were associated with the risk of ND.

Moreover, Liu et al., in a cross-sectional study including 5389 participants, showed that higher levels of oxidative balance score (OBS), a comprehensive indicator that considers various dietary components and lifestyle factors to assess an individual’s exposure to pro-oxidants and antioxidants, and dietary OBS were associated with a reduced risk of diabetic kidney disease, low-eGFR, and albuminuria, highlighting the importance of an antioxidant-rich diet and lifestyle among diabetic patients and suggesting that the integration of OBS into clinical practice may represent an innovative strategy to mitigate the burden of DN.

The management of DN is based on a holistic approach aimed at controlling blood glucose and blood pressure, reducing albuminuria, slowing disease progression and managing underlying comorbidities associated with DM.

If DN progresses to ESKD, the only available treatments are dialysis and kidney transplantation. However, neither significantly improves patient survival. As a result, increasing research efforts are focused on identifying new therapeutic targets and diagnostic markers.

At present, among the treatment available for DN, we find medications such as insulin or sodium-glucose transport protein 2 (SGLT2) inhibitors, renin-angiotensin-aldosterone system (RAAS) inhibitors and statins, which are often used to address dyslipidemia.

Interestingly, Guo et al. showed that long-term treatment with statins increased the risk of DN.

Zhou et al. clarify the role the potential targets of RAAS inhibitors for the treatment of DN.

Among cutting-edge treatments, nanotherapies are emerging as one of the most promising therapeutic approaches, as showed by (Paul et al.). In particular, nanotherapeutic platforms enable targeted drug delivery to renal tissues, improving therapeutic efficacy and reducing systemic side effects. This represents a significant advance over conventional therapies.

However, future research should focus on developing new treatments, particularly targeting inflammation, oxidative stress, and vascular dysfunction. Advances in technology will enable personalized and precision approaches of treatment.

In conclusion, this Research Topic underline that advances in biomarkers, innovative therapies and understanding the gut microbiota offer new hope to improve the quality of life of patients with DN. However, it is essential to promote longitudinal and multidimensional studies to validate these findings and translate them into personalized and effective clinical interventions.

Therefore, investing in translational research and implementing targeted prevention programs are essential. A comprehensive approach that integrates advanced therapies, lifestyle modifications, and continuous monitoring is crucial for effectively managing this complex pathology.

